# Development of a drug risk analysis and assessment system and its application in signal excavation and analysis of 263 cases of fluoroquinolone-induced adverse reactions

**DOI:** 10.3389/fphar.2022.892503

**Published:** 2022-10-04

**Authors:** Yuyao Guan, Lei Ji, Lei Zheng, Jing Yang, Yizhuo Qin, Ning Ding, Ting Miao, Xuemei Liu

**Affiliations:** ^1^ Department of Pharmacy, Shandong Provincial Third Hospital, Cheeloo College of Medicine, Shandong University, Jinan, China; ^2^ Shandong Provincial Key Laboratory of Applied Microbiology, Ecology Institute, Qilu University of Technology (Shandong Academy of Sciences), Jinan, China

**Keywords:** adverse drug reactions, drug risk analysis and assessment system, fluoroquinolones, signal detection, pharmacovigilance data mining

## Abstract

**Background:** Adverse drug reaction (ADR) signal mining is essential for assessing drug safety. However, the currently available methods for this are rather cumbersome.

**Objective:** We aimed to develop a drug risk analysis and assessment system using Java language and conduct pharmacovigilance data mining for fluoroquinolones at our hospital.

**Methods:** We used ADR data reported by Shandong Provincial Third Hospital between July 2007 and August 2021. The signal detection methods included proportional reporting ratio (PRR), reporting odds ratio (ROR), Bayesian Confidence Propagation Neural Network (BCPNN), Medicines and Healthcare products Regulatory Agency (MHRA). The BCPNN method was used as the reference standard for comparing the remaining three signal detection methods based on sensitivity, specificity, positive predictive value, negative predictive value, and Jorden index.

**Results:** The hospital database contained a total of 2,621 ADR reports, among which 263 were attributed to fluoroquinolones. There were 391 fluoroquinolone-ADR pairs. Using the PRR, ROR, MHRA, and BCPNN method, we detected 13 signals, 13 signals, 10 signals, and 11 weak signals, respectively. After signal detection, levofloxacin and moxifloxacin were shown to induce high risk signals for mental and sleep disorders, with the signal intensity of moxifloxacin being the most significant. Compared with BCPNN, the PRR and ROR methods showed better sensitivity, whereas the MHRA method showed better specificity.

**Conclusion:** We developed a drug risk analysis and assessment system that can help hospitals and other medical institutions to detect and analyse ADR signals in the self-reporting system database, and thus improve drug safety. Further, it indicates that the central nervous system damage caused by fluoroquinolones should be monitored closely, and thus provides a reference for the clinical application of these drugs.

## Introduction

Post-marketing drug safety supervision is directly related to public drug safety. Adverse drug reaction (ADR) signal mining plays an important role in assessing the post-marketing safety of drugs (Jones et al., 2001). ADR signal mining refers to the application of traditional epidemiological and statistical methods to describe and analyse the distribution of suspected drug use and effects (occurrence of adverse reactions) among drug users within a certain period, and then explore possible associations between the two (Peng et al., 2014). ADR signal detection is thus the most important technical work in ADR monitoring (Bate et al., 2002; Wilson et al., 2003). Using ADR signal detection technology to mine hospital ADR databases, identify risk signals in time, intervene the possible drug risk in time, and guarantee the safety of drug use in patients is of great significance to improve the quality of medical treatment. The Pharmacovigilance Quality Management Standard has been officially implemented in China since 1 December 2021. The Standard required medical institutions to carry out signal detection on collected adverse drug reactions and timely detect new drug safety risks.

Currently, adverse drug reaction signal mining methods include frequency-based and Bayesian methods. However, data processing requires professional software such as SPSS (Dai et al., 2012), R software (Shi et al., 2019), and SAS software package (Wei et al., 2019), which are highly professional and cumbersome to operate. Therefore, in this study, we developed a signal detection software, which only requires the user to import regular data into the software, and directly provides the corresponding data, signal detection results according to four algorithms, and the comparison results of these algorithms as the output.

Fluoroquinolones are used worldwide as they have good efficacy (Lee et al., 2018; Pasternak et al., 2018). However, surveillance data obtained after marketing fluoroquinolones has indicated an increase in serious adverse reactions. Epidemiological studies have reported an increased risk of rare adverse effects. These include tendinopathy and tendon rupture, peripheral neuropathy and aortic aneurysm (Baggio and Anand-Rajah., 2021). Therefore, in this study, pharmacovigilance data mining was performed for 263 cases of fluoroquinolone-associated ADR at Shandong Provincial Third Hospital using the self-designed drug risk analysis and assessment system.

## Materials and methods

### Data source

The data of adverse drug reactions reported by Shandong Provincial Third Hospital to the National Adverse drug Reaction Monitoring System from July 2007 to August 2021 were downloaded to conduct signal mining for the data of fluoroquinolone-induced ADR. To ensure a uniform standard for statistical analysis, the ADRs were coded according to the preferred terms (PT) of the Medical Dictionary for Drug Regulatory Activities (Med DRA).

### Data processing

The information regarding ADR reports was extracted from the database; uncertain and duplicate reports, as well as drug combination reports were excluded. As a single original ADR report may include various suspected drugs or ADRs, the data were split to obtain the corresponding drugs and ADR data. All ADR reports containing the generic names levofloxacin, moxifloxacin, and ciprofloxacin were included, along with original ADR reports indicating levofloxacin, moxifloxacin, and ciprofloxacin as suspected drugs.

### Research methods

In this study, the signal data-mining algorithm adopted the proportional reporting ratio (PRR), reporting odds ratio (ROR), Bayesian confidence propagation neural network (BCPNN), and Medicines and Healthcare products Regulatory Agency (MHRA) methods. Signal generation was conducted as per the conditions shown in Table 1.

**TABLE 1:** Signal data mining algorithms and their signal generating satisfied conditions.

Signal data mining algorithm	Signal generating satisfied conditions
Proportional reporting ratio (PRR)	PRR 95%CI > 1, and n ≥ 3, 1<PRR-1.96SE<50 weak signal (+); 50≤PRR-1.96SE<1 000 medium intensity signal (++); 1 000≤PRR-1.96SE high intensity signal (+++)
Reporting odds ratio (ROR)	PRR 95%CI > 1, and n ≥ 3, 1<ROR-1.96SE<50 weak signal (+); 50≤ROR-1.96SE<1 000 medium intensity signal (++); 1 000≤ROR-1.96SE high intensity signal (+++)
Medicines and Healthcare products Regulatory Agency (MHRA)	PRR ≥2, A ≥ 3, χ2 ≥ 4, 4≤χ2<100 weak signal (+); 100≤χ2<1 000 medium intensity signal (++); 1 000 ≤χ2 high intensity signal (+++)
Bayesian confidence propagation neural network (BCPNN)	IC95%CI lower limit >0, IC-2SD ≤ 0 no signal (−); 0 < IC-2SD ≤ 1.5 weak signal (+); 1.5 < IC-2SD ≤ 3.0 medium intensity signal (++); 3.0 < IC-2SD high intensity signal (+++)

Note: IC, information score; SD, standard deviation; CI, confidence interval.

False positive, false negative, true positive, and true negative were determined using the BCPNN method as the reference standard, and the sensitivity, specificity, positive predictive value, negative predictive value, and Jorden index were obtained to compare the other three detection methods. The calculation method is shown in Table 2.

**TABLE 2:** Four-fold table for calculating sensitivity and specificity.

	BCPNN (+)	BCPNN (−)	Total
Other method (+)	a	b	a+b
Other method (−)	c	d	c + d
Total	a+c	b + d	a+b + c + d

Note: sensitivity = a/(a+c); specificity = d/(b + d); positive predictive value = a/(a+b); negative predictive value = d/(c + d); Jorden index = [a/(a+c)]+[d/(b + d)]-1.

**SCHEME 1:**
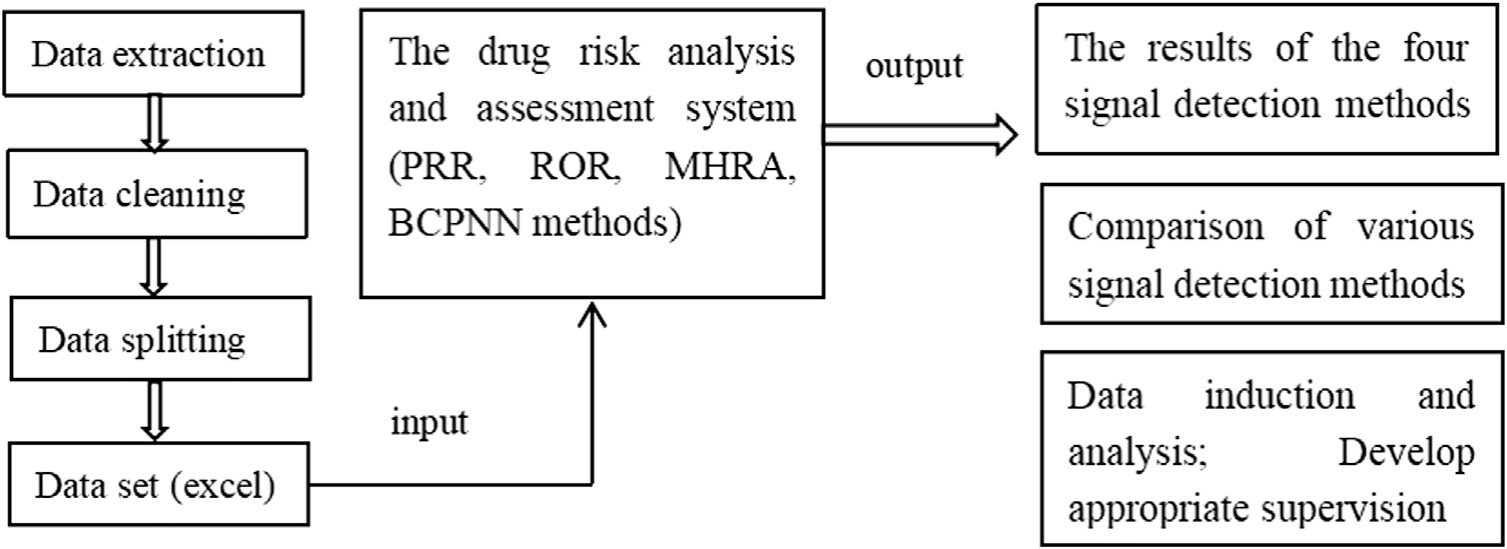
The protocol of the software procession

The web-based drug risk analysis and assessment system was developed using Java language. The code based on Java was written to achieve the bypass of parameters related to A, B, C, and D to obtain a complete detection data object. The results determined using the PRR, ROR, and MHRA methods were then compared with those obtained using the BCPNN method as the reference standard. Finally, the complete data and signal detection results were displayed on a web page in the form of data structure, which could be exported to an excel file. The protocol of system procession is shown in Scheme 1.

## Results

### ADR reporting and basic information of patients

In total, 2,621 ADR reports were downloaded from the National Adverse Drug Reaction Monitoring System, which were reported by Shandong Provincial Third Hospital during the study period; these included 181 cases of levofloxacin-induced ADR, 52 cases of moxifloxacin-induced ADR, and 30 cases of ciprofloxacin-induced ADR. Fluoroquinolones were the first suspected drug in 263 ADR cases, accounting for 10.03% of the total. Among these, there were 25 cases of levofloxacin, 4 cases of moxifloxacin, and 1 case of ciprofloxacin in patients with serious ADR, accounting for 11.41%. The cases of levofloxacin, moxifloxacin and ciprofloxacin in patients were respectively 72938, 11590, 5143. The incidence of levofloxacin, moxifloxacin and ciprofloxacin ADR were respectively 0.25%, 0.45%, 0.58% (see in Table 3). The patients included 118 male patients (44.87%) and 145 female patients (55.13%). Among 263 ADR cases, 36 patients (13.69%) were 18–40 years old, 96 (36.50%) were aged from 41 to 65 years, and 131 were >65 years old (49.81%). Cases of ADR in patients aged 18–40 years were less than those in the other two age groups, suggesting that ADR occurrence may be related to age.

**TABLE 3:** Adverse drug reactions (ADR) involving organs or systems and their main clinical manifestations.

Drugs	ADR Cases [%]	serious ADR Cases [%]	Cases of patients using drugs	Incidence of ADR
Levofloxacin	181(68.82%)	25(83.33%)	72938	0.0025
Moxifloxacin	52(19.77%)	4(13.33%)	11590	0.0045
Ciprofloxacin	30(11.41%)	1(3.33%)	5143	0.0058

Using Bio Portal tools (http://purl.Bioontology.org/ontology/MEDDRA), the signal corresponding to system organ classification (system organ class, SOC) was queried against the international medical terminology dictionary (MedDRA) terms; the ADRs involving organs or systems and main clinical manifestations are shown in Table 4.

**TABLE 4:** Signal detection results of adverse drug reactions with levofloxacin, moxifloxacin, and ciprofloxacin analysed using four signal mining algorithms.

ADRs involving organs or systems	Levofloxacin (cases)	Moxifloxacin (cases)	Ciprofloxacin (cases)	Main clinical manifestations
Skin and appendage disorders	119	33	21	Pruritus, rash, macules, red macules
Gastrointestinal system disorders	38	3	4	Diarrhoea, abdominal pain, abdominal distention, nausea, vomiting, hiccups
Body as a whole-general disorders	7	5	1	Chills, fever, anaphylaxis, anaphylactic shock, fatigue
Respiratory system disorders	22	3	2	Chest tightness, dyspnoea, wheezing, shortness of breath, laboured breathing
Central and peripheral nervous system disorders	40	13	8	Headache, dizziness, insomnia, mania, delirium, irritability, excitement, convulsions, numbness, tremors, auditory hallucinations, hallucinations
Cardiovascular and circulatory system disorders	41	17	5	Palpitation, nervousness, hypotension, hypertension, phlebitis, cyanosis, oedema, flushing, pallor, sweating
haematological system disorders	2	0	0	Leukopenia, thrombocytopenia
Metabolic and nutritional disorders	2	0	0	hypoglycaemia
Vision disorders	5	0	0	Eye itching, eye oedema, corneal detachment, abnormal vision

### ADR signal detection results

**FIGURE 1:**
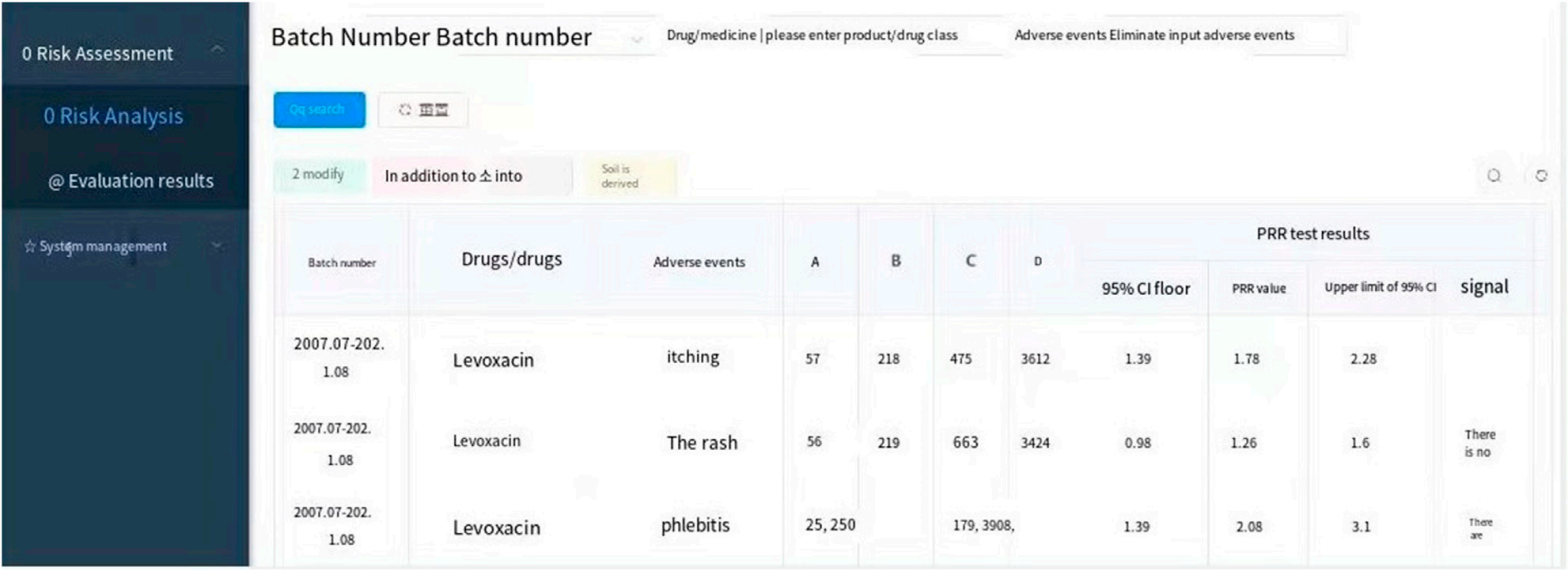
Output interface of the results and risk prompt of the four signal detection methods.

Among 263 ADR reports, there were 391 fluoroquinolone-ADR pairs. Adverse reactions with ≥3 reports were examined; we detected 13 weak signals using the PRR method, 13 weak signals using the ROR method, 10 weak signals using the MHRA method, and 11 weak signals using the BCPNN method. The original data were saved as excel files and imported into the drug risk analysis and assessment system, which automatically calculated the risk according to the four detection methods. The calculation results could be output on the web interface, and the results could be exported as excel files for saving. The detection results are shown in Figure 1 and in Table 5.

**TABLE 5:** Comparison of various signal detection methods (Drug-Adverse Events).

Generic name of medicine	ADR	Cases	PRR value of MHRA method	χ2 value of MHRA method	Signal results of MHRA method (Yes or No)	PRR value (95%CI)	Signal results of PRR method (Yes or No)	ROR value (95%CI)	Signal results of ROR method (Yes or No)	IC value of BCPNN method (95% CI)	Signal results of BCPNN method (Signal strength)
levofloxacin											
	pruritus	57	1.78	19.11	No	1.78 (1.39–2.28)	Yes	1.99 (1.46–2.70)	Yes	0.74 (0.30–1.18)	weak signal (+)
	rash	56	1.26	2.92	No	1.26 (0.98–1.60)	No	1.32 (0.97–1.79)	No	0.29 (-0.15–0.74)	No
	phlebitis	25	2.08	11.79	Yes	2.08 (1.39–3.10)	Yes	2.18 (1.41–3.38)	Yes	0.9 (0.28–1.51)	weak signal (+)
	nausea	19	0.96	0.0	No	0.96 (0.61–1.50)	No	0.96 (0.59–1.55)	No	-0.06 (-0.75–0.63)	No
	chest tightness	10	1.08	0.01	No	1.08 (0.58–2.04)	No	1.09 (0.57–2.09)	No	0.09 (-0.81–0.99)	No
	mental disorder	13	8.78	51.66	Yes	8.78 (4.47–17.24)	Yes	9.17 (4.57–18.41)	Yes	2.1 (1.29–2.90)	weak signal (+)
	vomiting	9	0.95	0.0	No	0.95 (0.49–1.84)	No	0.95 (0.48–1.88)	No	-0.08 (-1.02–0.87)	No
	sleep disorders	7	11.56	32.02	Yes	11.56 (4.34–30.80)	Yes	11.83 (4.37–32.02)	Yes	1.95 (0.90–2.99)	weak signal (+)
	dizziness	6	0.88	0.01	No	0.88 (0.39–1.99)	No	0.88 (0.38–2.02)	No	-0.16 (-1.28–0.95)	No
	papules	5	0.7	0.35	No	0.7 (0.29–1.70)	No	0.7 (0.28–1.72)	No	-0.43 (-1.63–0.77)	No
	abnormal defecate	5	1.38	0.18	No	1.38 (0.55–3.41)	No	1.38 (0.55–3.49)	No	0.32 (0.30–1.18)	No
	numbness	5	2.97	3.87	No	2.97 (1.15–7.70)	Yes	3.01 (1.14–7.92)	Yes	1.02 (-0.18–2.22)	No
	laboured breathing	4	1.32	0.06	No	1.32 (0.48–3.65)	No	1.33 (0.47–3.71)	No	0.27 (-1.05–1.58)	No
	abdominal discomfort	4	0.91	0.01	No	0.91 (0.34–2.49)	No	0.91 (0.33–2.53)	No	-0.12 (-1.43–1.19)	No
	hallucinations	4	11.89	16.21	Yes	11.89 (3.21–44.03)	Yes	12.05 (3.22–45.13)	Yes	1.62 (0.30–2.93)	weak signal (+)
	flushing	3	0.91	0.02	No	0.91 (0.29–2.90)	No	0.91 (0.28–2.93)	No	-0.12 (-1.58–1.34)	No
	convulsions	3	8.92	8.44	No	8.92 (2.14–37.12)	Yes	9.0 (2.14–37.88)	Yes	1.35 (-0.11–2.81)	No
	nervousness	3	0.31	4.09	No	0.31 (0.10–0.95)	No	0.3 (0.09–0.94)	No	-1.39 (-2.85–0.07)	No
	eye discomfort	3	1.35	0.03	No	1.35 (0.42–4.38)	No	1.35 (0.41–4.45)	No	0.26 (-1.20–1.72)	No
moxifloxacin											
	pruritus	19	2.07	10.3	Yes	2.07 (1.39–3.09)	Yes	2.41 (1.42–4.07)	Yes	0.93 (0.15–1.71)	weak signal (+)
	phlebitis	11	3.18	14.1	Yes	3.18 (1.80–5.60)	Yes	3.53 (1.84–6.79)	Yes	1.36 (0.42–2.31)	weak signal (+)
	rash	9	0.7	1.0	No	0.7 (0.38–1.30)	No	0.66 (0.33–1.34)	No	-0.48 (-1.49–0.53)	No
	mental disorder	5	11.25	30.45	Yes	11.25 (4.39–28.82)	Yes	11.94 (4.42–32.26)	Yes	1.95 (0.69–3.21)	weak signal (+)
	nervousness	5	1.77	0.99	No	1.77 (0.75–4.20)	No	1.82 (0.73–4.58)	No	0.62 (-0.64–1.88)	No
	sleep disorders	4	20.69	40.31	Yes	20.69 (6.63–64.57)	Yes	21.74 (6.67∼70.89)	Yes	1.96 (0.59–3.32)	weak signal (+)
	Papules	4	1.95	1.0	No	1.95 (0.74–5.17)	No	2.0 (0.72–5.58)	No	0.68 (-0.69–2.04)	No
	chest tightness	3	1.13	0.01	No	1.13 (0.37–3.48)	No	1.14 (0.35–3.65)	No	0.11 (-1.40–1.62)	No
ciprofloxacin											
	pruritus	13	2.28	8.96	Yes	2.28 (1.42–3.67)	Yes	2.75 (1.45–5.21)	Yes	1.02 (0.07–1.97)	weak signal (+)
	rash	7	0.88	0.03	No	0.88 (0.44–1.75)	No	0.86 (0.39–1.92)	No	-0.19 (-1.35–0.97)	No
	phlebitis	7	3.26	8.88	Yes	3.26 (1.62–6.57)	Yes	3.64 (1.61–8.21)	Yes	1.3 (0.13–2.46)	weak signal (+)
	nausea	4	1.13	0.0	No	1.13 (0.44–2.92)	No	1.15 (0.41–3.21)	No	0.12 (-1.29–1.52)	No

### Comparison of four signal detection methods

**FIGURE 2:**
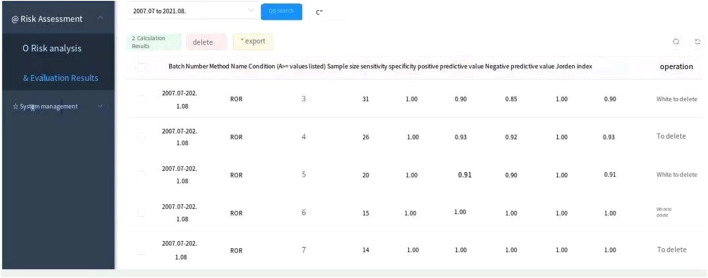
Output interface for comparing the results of the four signal detection methods.

**FIGURE 3:**
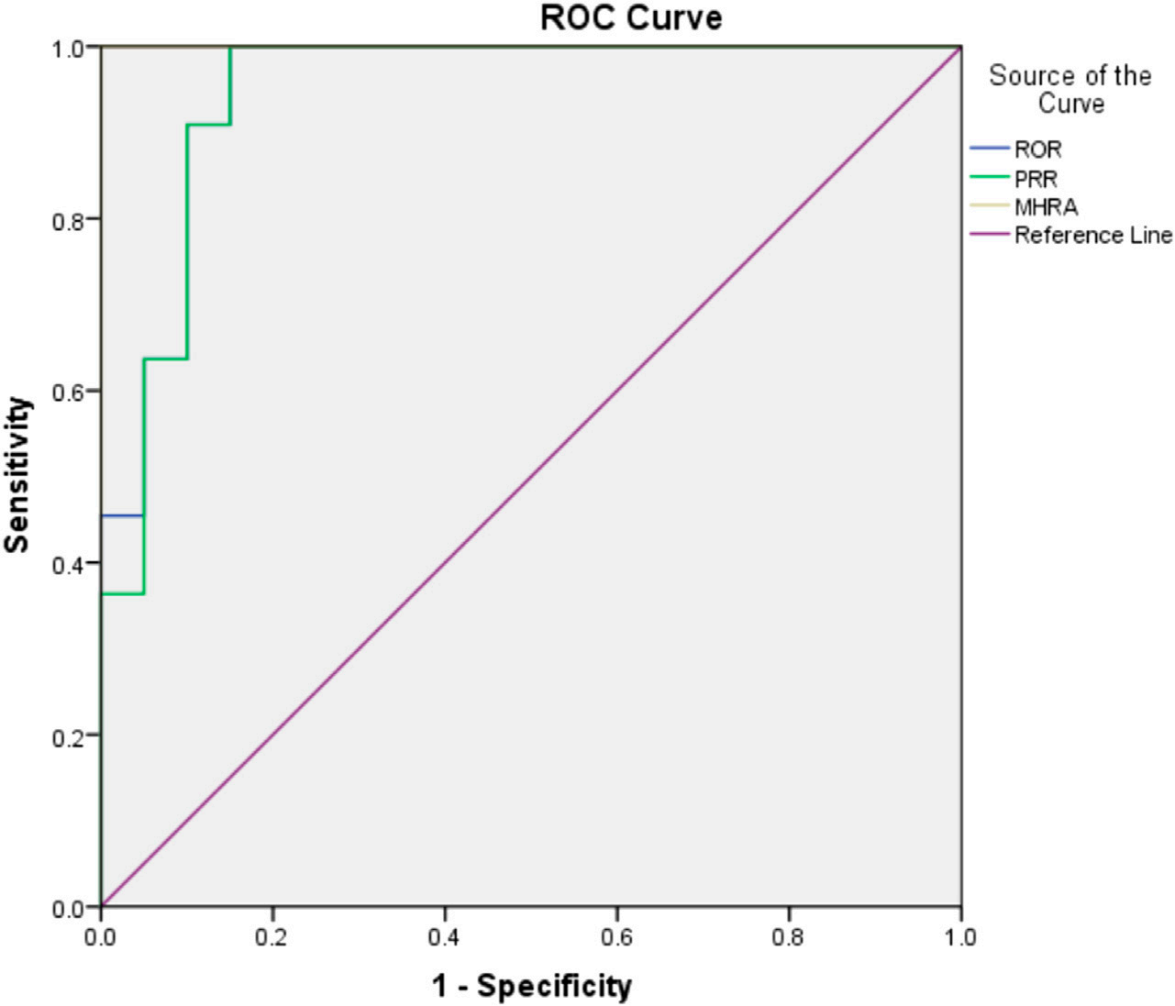
ROC curve of PRR, ROR and MHRA methods compared with BCPNN method.

The drug risk analysis and assessment system calculated the relative indicators for comparison across the imported fluoroquinolone data; the output results are shown in Figure 2 and Table 6. The comparison of the four methods depends on indicators of sensitivity, specificity, positive predictive value, negative predictive value and Jorden index. These indicators are calculated by comparing the values of the signals detected by the three methods with those detected by the BCPNN method. Receiver operating characteristic curve (ROC curve) was drawn with true sensitivity as ordinate and 1-specificity as abscissa (see in Figure 3). The area under the ROC curve of PRR, ROR and MHRA methods were 0.945, 0.950 and 1.000 respectively.

**TABLE 6:** Comparison of various signal detection methods (Drug-Adverse Events)

Method	Condition (A ≥ values listed)	Sample size	sensitivity	specificity	positive predictive value	negative predictive value	Jorden index
ROR	3	31	1.00	0.90	0.85	1.00	0.90
	4	26	1.00	0.93	0.92	1.00	0.93
	5	20	1.00	0.91	0.90	1.00	0.91
	6	15	1.00	1.00	1.00	1.00	1.00
	7	14	1.00	1.00	1.00	1.00	1.00
	11	8	1.00	1.00	1.00	1.00	1.00
PRR	3	31	1.00	0.90	0.85	1.00	0.90
	4	26	1.00	0.93	0.92	1.00	0.93
	5	20	1.00	0.91	0.90	1.00	0.91
	6	15	1.00	1.00	1.00	1.00	1.00
	7	14	1.00	1.00	1.00	1.00	1.00
	11	8	1.00	1.00	1.00	1.00	1.00
MHRA	3	31	0.91	1.00	1.00	0.95	0.91
	4	26	0.91	1.00	1.00	0.94	0.91
	5	20	0.89	1.00	1.00	0.92	0.89
	6	15	0.88	1.00	1.00	0.88	0.88
	7	14	0.88	1.00	1.00	0.86	0.88
	11	8	0.83	1.00	1.00	0.67	0.83

## Discussion

In this study, we designed a drug risk analysis and assessment system based on Java, which was simple and easy to operate. This system can realise the calculation and comparison of four signal detection methods. It can easily process ADR data and does not need expertise in complex data processing. When data are imported into the system, it can output the calculation results, risk signal identification, and detection method comparison results. Chen et al. developed similar system which only applied the BCPNN method and was not a web system, our system applied four methods including PRR, ROR, BCPNN, MHRA methods and can compare them (Chen et al., 2009). In future, this webpage version can be made available on the Internet, so that all hospitals can use this system to process and analyse ADR data, identify risk signals in a timely manner, strengthen medication supervision, and improve medication safety.

The PRR and ROR methods detected pruritus, phlebitis, psychosis, sleep disturbance, numbness, hallucination, and convulsion ADRs of levofloxacin; pruritus, phlebitis, psychosis, and sleep disturbance ADRs of moxifloxacin; and pruritus and phlebitis ADRs of ciprofloxacin as signals, which were all listed on the drug labels. In comparison, the MHRA method did not detect itch, numbness and convulsion ADRs of levofloxacin, and BCPNN method did not detect numbness and convulsion ADRs of levofloxacin as signals.

Overall, this study indicated that the central nervous system ADRs had high risk signals, including mental disorder and sleep disorder ADRs of moxifloxacin, and mental disorder, sleep disorder, illusion, and convulsion ADRs of levofloxacin; of these, the signal intensity of moxifloxacin was the most significant. Fluoroquinolones can enter the blood-brain barrier because of their high lipid solubility, and bind GABA receptors in competition with the central inhibitory neurotransmitter, γ-aminobutyric acid, thus increasing the excitability of the central nervous system. Central nervous system symptoms should thus be monitored closely in the clinical application of fluoroquinolones. Patients with renal insufficiency, neurological diseases, and elderly patients are more prone to severe central nervous system adverse reactions (Wasser et al., 2000). For patients with a history of epilepsy, Parkinson’s disease, psychiatric disorders, or familial disorders, these drugs should be avoided (Wingfield and Romero, 2001). To avoid serious mental symptoms in patients with renal insufficiency, especially in elderly patients, these drugs should be used as little or cautiously as possible; a reasonable administration plan should be formulated according to the patients’ physical conditions, the dose and time of administration should be adjusted, and the occurrence of adverse reactions in the central nervous system should be closely monitored (Samyde et al., 2016). Once neuropsychiatric symptoms are found, the medication should be stopped immediately (Li et al., 2019).

Tang et al. (2018) excavated and evaluated the post-marketing safe warning signals of fluoroquinolones from FAERS submitted to FDA with ROR method. Fluoroquinolones were shown high risk signals of severe tendon and ligament injuries, such as tendon rupture, tendonitis, tenosynovitis, ligament rupture, tendon pain, etc. The data sample size of this study was large, reflecting the overall risk of fluoroquinolones. Our study can reflect the risk of fluoroquinolones in our hospital, and can help us take targeted preventive measures.

Compared with BCPNN, the PRR and ROR methods showed better sensitivity, whereas the MHRA method had better specificity. Higher sensitivity resulted in a lower false negative rate (missed diagnosis rate), and higher specificity resulted in a lower false positive rate (misdiagnosis rate). The area under the ROC curve of PRR, ROR and MHRA methods were greater than 0.9, which indicates that the three methods have good risk signal recognition. Using our drug risk analysis and assessment system, the advantages of the four detection methods can be integrated to systematically analyse the detection results.

### Study limitations

The separation and aggregation of ADR data was processed by manual. In the future, we hope to expand the functions of the software to achieve automatic processing.

## Conculsions

Through this drug risk analysis and assessment system, the adverse reaction data reported by our hospital can be analysed in a timely manner. Further, its operation is simple and easy, and can be made available on the Internet in future, so that additional hospitals can analyse the risks of adverse drug reactions, adopt appropriate supervision, and improve drug safety. In this study, four kinds of signal detection methods were used to analyse the safety of fluoroquinolones, which indicating a high risk of central nervous system damage, which provides a reference for the clinical application of these drugs. In future, we will carry out signal detection for other drug adverse reactions in our hospital and Jinan Adverse drug Reaction Monitoring Center.

## Data Availability

The original contributions presented in the study are included in the article/supplementary material, further inquiries can be directed to the corresponding authors.
